# Transcriptomics of Meiosis in the Male Mouse

**DOI:** 10.3389/fcell.2021.626020

**Published:** 2021-03-05

**Authors:** Adriana Geisinger, Rosana Rodríguez-Casuriaga, Ricardo Benavente

**Affiliations:** ^1^Biochemistry-Molecular Biology, Facultad de Ciencias, Universidad de la República (UdelaR), Montevideo, Uruguay; ^2^Department of Molecular Biology, Instituto de Investigaciones Biológicas Clemente Estable (IIBCE), Montevideo, Uruguay; ^3^Department of Cell and Developmental Biology, Biocenter, University of Würzburg, Würzburg, Germany

**Keywords:** meiosis, transcriptomics, RNAseq, meiotic prophase, spermatogenesis, lncRNAs, MSCI, spermatogenic cell sorting

## Abstract

Molecular studies of meiosis in mammals have been long relegated due to some intrinsic obstacles, namely the impossibility to reproduce the process *in vitro*, and the difficulty to obtain highly pure isolated cells of the different meiotic stages. In the recent years, some technical advances, from the improvement of flow cytometry sorting protocols to single-cell RNAseq, are enabling to profile the transcriptome and its fluctuations along the meiotic process. In this mini-review we will outline the diverse methodological approaches that have been employed, and some of the main findings that have started to arise from these studies. As for practical reasons most studies have been carried out in males, and mostly using mouse as a model, our focus will be on murine male meiosis, although also including specific comments about humans. Particularly, we will center on the controversy about gene expression during early meiotic prophase; the widespread existing gap between transcription and translation in meiotic cells; the expression patterns and potential roles of meiotic long non-coding RNAs; and the visualization of meiotic sex chromosome inactivation from the RNAseq perspective.

## Introduction

The alteration of the meiotic program is at the basis of an important number of fertility problems (Handel and Schimenti, 2010; Hann et al., 2011; Geisinger and Benavente, 2016; Gheldof et al., 2019; Veitia, 2020) and other pathologies (e.g., Tsui and Crismani, 2019), including cancer (Feichtinger and McFarlane, 2019). Therefore, the need to improve the knowledge on the molecular groundwork of meiosis in mammals is obvious. However, the studies on the molecular bases of mammalian meiosis have been hampered by some intrinsic obstacles. In the first place, the lack of reliable and robust *in vitro* culture systems of mammalian meiotic cells (Handel et al., 2014; Sun et al., 2014; Komeya et al., 2018; Hayashi, 2019; Bharti et al., 2020) is an important drawback that raises the need to work with *in vivo* models. Besides, in females the main meiotic events take place during the embryonic phase, and the number of oocytes is scarce, which hinders molecular analyses (e.g., Hernández-López et al., 2020). Studies in males are more accessible as meiosis starts in the postnatal life, and the testes produce massive numbers of gametes; consequently, most studies have been performed in males. Notwithstanding this, male meiosis is part of the asynchronous and continuous spermatogenic process (Griswold, 2016), and therefore germ cells spanning all the different stages of spermatogenesis simultaneously coexist within adult testes, altogether with different types of testicular somatic cells. Testicular heterogeneity constitutes a challenge when trying to unravel the molecular program of a specific stage or cell type, as a pre-requisite is the availability of methods to allow profiling that specific cell type separately from the whole mix. Particularly, pachytene spermatocytes (PS) comprise about 5% in adult mice testicular cell suspensions (Soumillon et al., 2013, and our own observations).

As mouse is the most popular mammalian model because of its relatively easy maintenance and manipulation, in addition to its highly curated genome, most transcriptomic studies on mammalian meiosis have been carried out in mice. Nevertheless, despite the extensive similarities with human meiosis, it must be recalled that some notorious between-species differences exist, including the histological organization of the testis, duration of the seminiferous epithelium cycle, and germline-niche interactions, among others (Guo et al., 2018, 2020; Shami et al., 2020). This being said, mouse studies are significantly contributing to increase our understanding about human meiosis and its associated pathologies.

Microarray-based studies have been employed for profiling the transcriptome along spermatogenesis (e.g., Schultz et al., 2003; Maratou et al., 2004; Rossi et al., 2004; Shima et al., 2004; Pang et al., 2006; Chalmel et al., 2007; Fallahi et al., 2010; Waldman Ben-Asher et al., 2010; Bao et al., 2013; Sun et al., 2013; Liang et al., 2014), albeit these are being largely replaced by RNAseq due to its increased sensitivity, and to its ability to identify previously unknown transcripts and novel isoforms (Roy et al., 2011; Mutz et al., 2013).

In this mini-review, we will outline some interesting features that are starting to arise from transcriptomic studies of murine meiotic cells, mostly based on RNAseq (although some microarray results will be also included). As due to the above-mentioned constraints the vast majority of reports correspond to males, we will focus on male meiosis. A number of recent studies have addressed the analysis of gene expression in mouse meiotic precursor cells, to evaluate spermatogonial differentiation and/or mitosis-to-meiosis transition (Green et al., 2018; Hermann et al., 2018; La et al., 2018; Ernst et al., 2019; Grive et al., 2019; Law et al., 2019; Liao et al., 2019; Velte et al., 2019; Tan et al., 2020). Here, due to space limitations, we will only focus on the meiotic phase itself. Overall, transcriptomic analyses have shown that male meiotic (and post-meiotic) cells have an extremely complex transcriptome (Soumillon et al., 2013), expressing a panoply of mRNAs and splice variants, long non-coding RNAs (lncRNAs), and small non-coding RNAs (sncRNAs). Far from aiming to cover all the knowledge in the field, our objective is to illustrate how the methodologies that allow to isolate different specific meiotic cell populations or to profile individual cells, in combination with transcriptomic techniques, are contributing to our understanding of the molecular bases of meiosis. In particular, we will center on the different approaches that have been employed to enable profiling the transcriptome of isolated/individual murine meiotic cells, and on some novel aspects we have selected to develop, specifically concerning coding genes and lncRNAs. On the other hand, sncRNAs – including miRNAs and piRNAs – play essential roles for the control of meiosis and spermiogenesis progression (e.g., Gou et al., 2014; Goh et al., 2015; Dai et al., 2019), and certainly would deserve a chapter. However, as exhaustive reviews about them have been published elsewhere, and in order not to extend further, we will not elaborate on the subject here (for revisions on meiotic sncRNAs, see Bortvin, 2013; de Mateo and Sassone-Corsi, 2014; Kotaja, 2014; Yadav and Kotaja, 2014; Wang and Xu, 2015; Luo et al., 2016; Dai et al., 2020, among others).

## Strategies for the Obtainment of Meiotic Cells for RNAseq

Different studies have addressed the complexity of mammalian testicular gene expression through RNAseq, by analyzing bulk RNA from testes of pre-pubertal animals at increasing ages along the semi-synchronous first spermatogenic wave, where the new transcripts are attributed to the newly appeared cell types (e.g., Gong et al., 2013; Laiho et al., 2013; Weng et al., 2017). The disadvantages of these studies are that they do not allow undoubtedly assigning specific RNAs to a certain cell type, and fail in the characterization of transcripts from poorly represented cell types such as those from early meiotic prophase stages. Besides, they do not take into account the intricate cell-cell interactions within the testis (e.g., between spermatogenic and somatic Sertoli cells), where some cell types can change their expression patterns in contact with the newly appeared cell types (Green et al., 2018; Guo et al., 2020; Tan et al., 2020). As an attempt to partially overcome these limitations, some studies have combined the use of testes of individuals at increasing ages with a computational approach, in order to de-convolve the temporal expression profiles into cell type-specific expression profiles (Margolin et al., 2014; Ball et al., 2016).

The alternative approach has been the use of isolated testicular cell populations or (more recently) individual cells, to more accurately profile the transcriptome of specific stages along the spermatogenic process. Beyond the fact that differences between the first spermatogenic round and the following ones have been reported (Yoshida et al., 2006; Grive et al., 2019), either adult or pre-pubertal individuals at increasing ages have been used. Historically, the most classical methods for obtaining stage-specific spermatogenic cell populations from rodent testicles, have been STA-PUT (i.e., a gravimetric decantation in an albumin gradient; Lam et al., 1970; Go et al., 1971; Romrell et al., 1976; Bellvé, 1993) and centrifugal elutriation (Meistrich, 1977). Both methods have been employed for the obtainment of enriched cell populations for RNAseq (e.g., Gan et al., 2013b; Soumillon et al., 2013; Chalmel et al., 2014; Hammoud et al., 2014; Sin et al., 2015; Lin et al., 2016; Wichman et al., 2017). However, these methods only allow the obtainment of highly enriched but not pure cell populations (Meistrich, 1977; Soumillon et al., 2013), and only of certain specific cell types, while other cell types are obtained at low purity levels (Meistrich, 1977). Particularly concerning meiosis, PS have been mostly taken as the representative meiotic stage, as they constitute the only meiotic cell type that can be obtained with a significant enrichment because of their relative abundance and larger size/density (Meistrich, 1977).

Multi-parametric flow cytometry (FCM) has been used to analyze and sort different testicular cell populations based on DNA content together with differences in nuclear size, cellular size, complexity, and chromatin compaction (Geisinger and Rodríguez-Casuriaga, 2010). Besides the advantage of enabling the obtainment of highly pure stage-specific cell populations, it allows the discrimination and eventual classification of a higher number of cell populations (Malkov et al., 1998; Bastos et al., 2005). Taking advantage of the blue and red fluorescence of the vital dye Hoechst 33342 (Bastos et al., 2005; Getun et al., 2011), Fallahi et al. (2010) isolated spermatogonia, pre-leptotene (pre-L), leptotene-zygotene (L/Z), early-PS, middle-PS, late-PS, diplotene (D), and round spermatids (RS) from adult male mice with over 95% purity for each fraction, by fluorescence activated cell sorting (FACS). The sorted cell populations were used for transcriptome profiling by microarray analysis (Fallahi et al., 2010). In our laboratory, we have developed a protocol for the purification of testicular cell populations by FACS using Vybrant DyeCycle Green, a non-cytotoxic vital dye with the advantage over Hoechst 33342 that it is excited with a blue laser thus avoiding the need of a UV laser, which in turn minimizes potential damage to nucleic acids caused by UV light exposure (Rodríguez-Casuriaga et al., 2014; Geisinger and Rodríguez-Casuriaga, 2017). This protocol allowed the profiling of coding transcripts and lncRNAs along spermatogenesis through RNAseq, using highly pure (>95%) stage-specific cell populations (da Cruz et al., 2016; Trovero et al., 2020). These studies included the L/Z cell population, thus enabling for the first time to compare the transcriptomic profiles, as obtained through NGS, between early and medium/late meiotic prophase, and providing information on gene expression fluctuations along meiotic prophase (da Cruz et al., 2016). An optimized Hoechst-33342-based FCM protocol for sorting enriched leptotene (L) aside from zygotene (Z) cell populations (60–80% and 75–90% purity, respectively) from adult mouse testis has been described (Gaysinskaya et al., 2014) and used for genome-wide methylation analyses (Gaysinskaya et al., 2018), although no extensive transcriptome profiling studies using this protocol have been reported so far.

Fluorescence activated cell sorting can be combined with antibodies to sort additional cell types, mainly specific populations of spermatogonia. As an example, Zhu et al. (2016) used a combination of FACS and Magnetic Activated Cell Sorting (MACS) to classify spermatocytes, spermatids, and undifferentiated spermatogonia from human testicular biopsies, for RNAseq. Another strategy for generating germ cell-specific transcriptome profiles from human biopsies, where the amount of material is very limiting, has been individual-cell laser capture microdissection (LCM). This method allowed the selection and transcriptome profiling of six distinct germ cell subtypes based on morphology, location in the seminiferous tubular cross-section, and germ cell associations at the various stages of the seminiferous epithelium; concerning meiotic cells, L/Z, early PS, and late PS were profiled (Jan et al., 2017).

A completely different approach has been the use of “meiotic-like” immortalized mouse cell lines. GC-1 cells (American Type Culture Collection [ATCC], Manassas, VA, United States) were created by transformation of type B spermatogonia with pSV3-neo, and are claimed to exhibit the characteristics of a stage between type B spermatogonia and primary spermatocytes, while GC-2 cells (ATCC) originated by transformation of spermatocytes with SV40 large T antigen, are arrested at a pre-meiotic stage, and are claimed to exhibit the characteristics of primary spermatocytes. A couple of reports compared the transcriptomes of these cell lines (Zhang et al., 2013; Hong et al., 2018). Notwithstanding, to what extent their transcriptomic profiles resemble those of pre-meiotic and meiotic prophase cells, is highly doubtful. In fact, a microarray comparison with whole testis profiles showed that a very small proportion of the testis-specific mRNAs and lncRNAs were detected in these cell lines (Hong et al., 2018), thus indicating that they have a limited usefulness.

Recently, single cell RNA sequencing (scRNAseq) has gained much attention, as it enables to profile the transcriptome of thousands of single cells in a population. Pseudotime ordering allows arranging cells along a continuous path that represents the evolution of a process. Thus, scRNAseq permits to capture the continuity of spermatogenesis, rather than artificially chosen stages. Moreover, it allows characterize the existing heterogeneity at any given phase, as well as the RNA content of rare cell populations (Suzuki et al., 2019; Soraggi et al., 2020). Various laboratories have addressed the characterization of RNA fingerprints from human testicular samples (Guo et al., 2018; Hermann et al., 2018; Wang et al., 2018; Shami et al., 2020) and mouse testes (Green et al., 2018; Hermann et al., 2018; Lukassen et al., 2018; Ernst et al., 2019; Grive et al., 2019; Jung et al., 2019) through scRNAseq (for a revision on the different used methodologies for scRNAseq and data-analysis, see Suzuki et al., 2019; Soraggi et al., 2020). In general, these studies coincide that spermatogenesis progresses as a continuum, with no clear-cut changes between the transcriptomes of successive cell types (Green et al., 2018; Guo et al., 2018; Hermann et al., 2018; Lukassen et al., 2018; Jung et al., 2019). One aspect to note, is that while in the methods relying on the isolation of cell populations cell type-assignment is generally based on the analysis of an aliquot of each sorted population (microscopical observation, immune labeling for cell type-specific protein markers), in unsorted scRNAseq studies deduction of cell type/subtype is done through the expression of marker genes. However, due to the pronounced uncoupling between transcription and translation along spermatogenesis (see below), we consider that this criterion may be misleading for staging purposes. For a more accurate cell-type assignment, some have compared the expression profiles of single cells from total testis dissociation, to those of stage-specific cells purified by FACS or STA-PUT (Hermann et al., 2018; Jung et al., 2019) or to available data sets from isolated cells of known identity (e.g., Ernst et al., 2019).

Chen et al. (2018) combined transgenic labeling by means of Vasa-dTomato (expressed in spermatogenic cells) and Lin28-YFP (expressed in undifferentiated spermatogonia), with synchronization of the cycle of the seminiferous epithelium using WIN 18,446/retinoic acid (Hogarth et al., 2013) for FACS sorting and scRNAseq. This allowed the profiling of 20 different cell subtypes from synchronized testicular tissues. Thus far this has been the most complete RNAseq study in isolated cells, as it included some previously unpurified cell types. Concerning meiotic cells, L, Z, early-PS, middle-PS, late-PS, D, metaphase I, and metaphase II were profiled (Chen et al., 2018). Anyway, it must be recalled that spermatogenesis was manipulated, and although no overt differences in appearance, fertility, or gene expression between synchronized and unsynchronized testes have been detected (Romer et al., 2018), some gene expression differences with the normal, asynchronous process cannot be excluded.

Altogether, despite the diversity of approaches and methodologies, the different studies are allowing to reach some common conclusions that shed light on the transcriptomic landscape of the complex meiotic process. On the other hand, in some other cases results are not conclusive as different studies have reached controversial results, leaving the questions remain open. In the next sections we will go over only a few of the many interesting aspects that are emerging from these studies, trying to highlight some of the common findings, as well as some of the controversial points where more research is still needed.

## Gene Expression in Early Meiotic Prophase

Early studies measuring the incorporation of [H^3^]uridine or [H^3^]cytidine had suggested null (Monesi, 1964) or very low (Kierszenbaum and Tres, 1974) transcription levels in mouse testes during early meiotic prophase (i.e., L and Z), and even during early pachytene (P), in comparison to later prophase stages. This was supported by Page et al. (2012), who observed low levels of RNA polymerase II and the active-chromatin marker H3K9ac (histone H3 acetylated at lysine 9) in L, Z, and early PS, but a strong increase in mid-PS, while the marker for gene silencing and heterochromatin H3K9me3 (histone H3 trimethylated at lysine 9), showed the opposite pattern.

As noted above, transcriptomic studies in the short L and Z stages have been long relegated compared to those in PS. Now, experiments including isolated early meiotic prophase cells are allowing to address gene expression during L/Z. In general, the different reports agree that murine early spermatocytes present a low complexity transcriptome (Chen et al., 2018; Ernst et al., 2019), and this would stand true for humans as well (Jan et al., 2017). In spite of that, the power of RNAseq technology in combination with the use of isolated L/Z spermatocytes, reveal that important genes are differentially expressed during early meiotic prophase.

Using a highly pure L/Z cell population, we have shown the existence of a set of genes that are upregulated in L/Z, with almost half of them exhibiting a marked expression peak in these early stages. This group of genes that peak in L/Z, to decay before the P stage, includes genes related to meiotic processes (gene ontology [GO] terms “meiosis,” “synaptonemal complex,” “meiotic recombination,” “chromosome condensation,” “meiotic chromosome segregation”), among others (da Cruz et al., 2016). The study by Chen et al. (2018), whose 20 different isolated spermatogenic cell types allowed very fine discrimination, also showed an expression peak during early prophase, and a decrease in PS, for the representative terms “meiotic DNA double-strand break formation” and “meiotic chromosome segregation.” Moreover, differentially expressed genes in the categories “meiotic cell cycle,” “DNA repair,” and “DNA recombination,” appeared downregulated after L/Z (Chen et al., 2018). This would be also the case for human testes, where the expression of genes involved in meiotic recombination, homologous synapsis, synaptonemal complex, and compaction of chromatin, were detected as upregulated in L/Z (Jan et al., 2017; Wang et al., 2018). Besides, germ cell-specific transcriptomic profiles indicated that the genes contained within the GO terms “reciprocal meiotic recombination” and “meiotic chromosome segregation” were highly conserved between mouse and human, and mainly expressed in human L/Z spermatocytes (Jan et al., 2017).

Of course, it is very likely that at least some of the transcripts we detect with a marked expression peak in L/Z were already present in a sub-population of spermatogonia/pre-L cells. In fact, different studies indicate that the commitment to meiosis takes place in pre-meiotic cells, with meiotic genes being turned on before meiosis onset (e.g., Evans et al., 2014; Jan et al., 2017; Chen et al., 2018), and it seems that this could be the case for female meiosis as well (Soh et al., 2015). Anyway, transcriptomic studies make it clear that a group of genes related to male meiotic processes present an expression peak during early prophase, to decay later on in PS.

## The Lag Between Transcription and Translation

Post-transcriptional regulation and particularly translational delay are a hallmark of spermatogenesis; their massiveness can be evidenced through transcriptome profiling.

As stated above, we and others have reported an expression peak in L/Z for genes within GO terms related to early meiotic events, but also for others related to late meiotic events such as “meiotic chromosome segregation,” both for mouse (da Cruz et al., 2016; Chen et al., 2018) and human (Jan et al., 2017). Furthermore, many of the significantly expressed genes within those categories, were already up in pre-L (Chen et al., 2018). Curiously, some genes whose protein products play their roles in post-meiotic stages, are also differentially expressed in L/Z (da Cruz et al., 2016), or even earlier. These include genes coding for proteins related to sperm function and motility such as *Hspa5*, *Tex101*, *Ly6k* (da Cruz et al., 2016), *Odf2*, *Cabyr*, *Tcp11*, and *Hook1* (Jan et al., 2017), to name a few. Notably, an RNAseq analysis of mice mutant for *Prdm9* (that encodes a histone methyltransferase expressed in L/Z and required for the activation of recombination), showed that although the mutant testes were cytologically arrested in a late-L/Z stage, they nevertheless developed gene expression signatures characteristic of later developmental substages (Fine et al., 2019).

**FIGURE 1:**
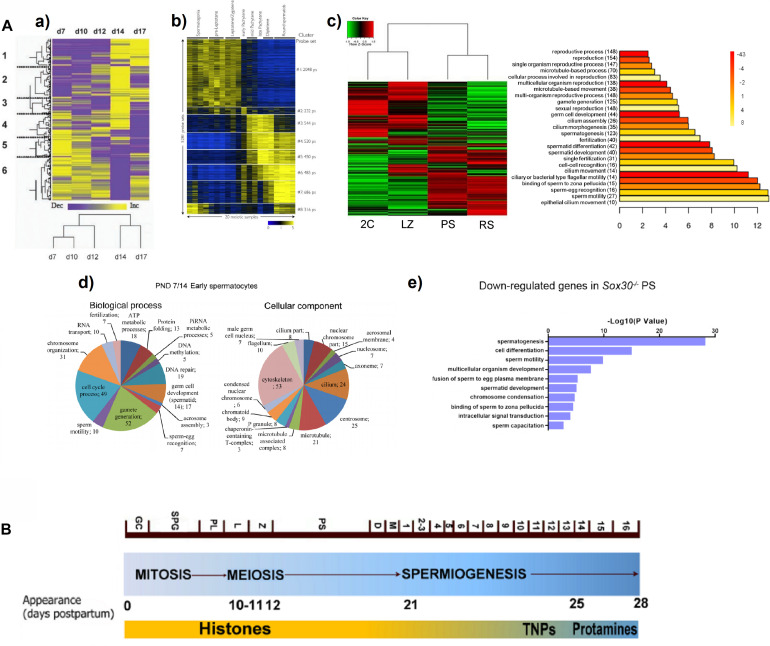
**(A)** Examples from reports showing a switch in gene expression patterns, and the expression of spermiogenesis-specific programs during mouse meiotic prophase. **(a)** Heat map representing the clustering of 790 differentially expressed sequences derived from a microarray study in testes of animals of increasing age, from 7 days post-partum (d7) to 17 days post-partum (d17). Columns represent the expression profile of all the sequences for the indicated post-natal ages, while rows represent expression of specific genes over time (low expression levels are shown in purple, and high expression levels in yellow). Note the overall change from d12 to d14, coinciding with pachytene onset. A tree representing clustering by resemblance of expression profiles across the indicated post-natal ages is shown below. The figure is reproduced from Waldman Ben-Asher et al. (2010), with permission from John Wiley and Sons (license number 5014730359883). **(b)** Heat map showing the relative expression levels of 5,281 microarray probe sets divided into 8 K-means clusters, in different spermatogenic cell populations purified by FACS. Each horizontal line corresponds to a probe set (high expression in yellow, low expression in blue). The switch in the transcriptome between L/Z and mid-P is evident. Reprinted from Fallahi et al. (2010), under the Creative Commons Attribution License. **(c)** Heat map of expression levels and hierarchical clustering for the global differential gene expression in four FACS-sorted spermatogenic cell populations, profiled through RNAseq (2C, spermatogonia and somatic testicular cells; LZ, leptotene/zygotene; PS, pachytene spermatocytes; RS, round spermatids). High expression: red; low expression: green. To the right, the main enriched GO terms for biological process category (BP) of the upregulated genes in PS are shown. The heat map evidences a switch in gene expression patterns from L/Z to PS, while the GO analysis shows enrichment in spermiogenesis-related terms. Reproduced from da Cruz et al. (2016), under the Creative Commons Attribution License. **(d)** Pie charts showing enriched BP and cellular component (CC) GO terms among upregulated genes in the testes of animals at post-natal day 14 compared to those of post-natal day 7 (PND7/14), obtained from an RNAseq study. Note the upregulation of spermiogenesis-related terms both for BP and CC categories. The figure is reproduced from Laiho et al. (2013), under the Creative Commons Attribution License. **(e)** GO term enrichment analysis for downregulated transcripts in PS of *Sox30*^–/–^ mice compared to WT, as assessed through RNAseq of STA-PUT-isolated stage-specific spermatogenic cells. Reprinted from Bai et al. (2018), with permission from The Company of Biologists Ltd.; permission conveyed through Copyright Clearance Center, Inc. **(B)** Diagram representing the three main spermatogenic phases, and the timing of mouse spermatogenesis. The different cell types and the onset of some of them (days postpartum) are indicated. The substitution of histones – first by transition proteins (TNPs) and then by protamines – is shown as well. GC, gonocytes; SPG, spermatogonia; PL, preleptotene; L, leptotene; Z, zygotene; PS, pachytene; D, diplotene; M, meiotic divisions; 1–16 represent the different spermatid stages. Adapted from Trovero et al. (2020), under the Creative Commons Attribution License.

Despite the uncoupling between transcription and translation in PS is known, especially striking is the finding that the P transcriptome reveals widespread early expression of genes related to post-meiotic processes. Concurring with a couple of previous microarray studies (Fallahi et al., 2010; Waldman Ben-Asher et al., 2010; Figures 1A,a,b), our RNAseq analyses detected a global expression switch in the testicular transcriptome during the progression from Z to P (da Cruz et al., 2016; Figure 1A,c). Moreover, this switch coincides with the turning off of a high number of meiotic genes, and the turning on of spermiogenesis-related ones (da Cruz et al., 2016). Specifically, terms related to “spermatid differentiation and development,” “fertilization,” “cilium and flagellum assembly and motility,” “sperm-egg recognition,” and “binding of sperm to zona pellucida,” were among the most significantly represented GO categories within the differentially expressed genes at the L/Z-to-P transition (da Cruz et al., 2016; see Figure 1A,c), thus indicating that the spermiogenesis programs are turned on as early as during meiotic prophase. This is consistent with the results from Soumillon et al. (2013), who found that a gene cluster strongly upregulated in spermatocytes and spermatids, was significantly enriched with genes involved in spermatogenesis, gamete generation, sperm motility, and fertilization. Similar results were reported in the scRNAseq study from Jung et al. (2019). Also coinciding with other reports, Hermann et al. (2018) observed that although the expression of some genes for products of the spermatozoon such as sperm-specific glycolytic isozymes (*Gapdhs*, *Ldhc*, *Pgk2*, etc.) peaks in RS, their transcription starts in primary spermatocytes. Even studies using whole testes of individuals of increasing ages, either with deconvolution (Ball et al., 2016) or without it (Laiho et al., 2013), showed an enrichment in the expression of genes related to microtubule and cilia, spermiogenesis, and fertilization, at ages corresponding to the appearance of P/D spermatocytes (Figure 1A,d). The expression of sperm-related genes in meiotic prophase, has been also reported for men (Jan et al., 2017; Hermann et al., 2018).

It is interesting that a transcriptome analysis of STA-PUT–fractionated cells from *Sox30*^–/–^ infertile mice, revealed SOX30 as a testis-specific transcription factor essential for activating haploid differentiation programs during the later stages of meiotic prophase. Loss of SOX30 resulted in the downregulation of a set of genes in PS (Figure 1A,e), and these downregulated genes were related to spermatogenesis, spermatid development, sperm motility, fusion of sperm to egg plasma membrane, and sperm capacitation (Bai et al., 2018).

In the same line of evidence, a microarray study showed that loss of A-MYB (MYBL1) – a male-specific master regulator of several meiotic processes that is expressed in PS (Bolcun-Filas et al., 2011) and is essential for the production of piRNAs and piRNA-pathway proteins (Li et al., 2013) – not only results in misregulation of meiotic genes, but also in the downregulation of genes whose transcripts are translated post-meiotically and involved in post-meiotic functions (Bolcun-Filas et al., 2011). A novel finding indicates that A-MYB is a key regulator of meiotic super-enhancers (i.e., regions of the genome comprising multiple enhancers that regulate important genes for cell identity) (Maezawa et al., 2020). Interestingly, many genes that are adjacent to meiotic super-enhancers and would be regulated by A-MYB, are known to be critical for late spermatogenesis (Maezawa et al., 2020). One such critical genes is *PIWIL1*, for which a human transcriptomic study indicated that its expression peaks in PS (Jan et al., 2017), and whose product, besides functioning in transposon silencing, has been shown to exert a role in the post-transcriptional regulation of mRNAs encoding spermatid-specific proteins (e.g., Gou et al., 2014; Dai et al., 2019).

The switch in gene expression programs could be facilitated by genome-wide changes leading to the *de novo* formation of accessible chromatin (Maezawa et al., 2018), and the extensive reprogramming of chromatin 3D architecture that takes place in meiotic cells (Alavattam et al., 2019; Patel et al., 2019; Vara et al., 2019; Wang et al., 2019; Luo et al., 2020). An integration of Hi-C (high-throughput genome-wide chromatin conformation capture) and RNA-seq from purified mouse spermatogenic cell populations, showed a switching in a subset of B (inactive) compartments to A (active) compartments in the chromatin of PS; the number of genome regions in A compartments was higher in PS, suggesting that PS chromatin is in a more transcriptionally active state. Notably, a number of genes that were originally located in compartment B regions in primitive spermatogonia and switched to compartment A regions in PS, and at same time increased their expression, have a function in DNA DSBs repair, but also in cilium formation, critical for normal sperm physiology (Luo et al., 2020).

The temporal uncoupling between transcription and translation in testicular germ cells, would be related to the need for extensive post-transcriptional regulation. During the last stages of spermiogenesis, histones are sequentially replaced first by transition proteins and then by protamines (Figure 1B), which results in transcriptional silencing (e.g., Braun, 1998; Kleene, 2001; Legrand and Hobbs, 2018). Extensive early transcription and RNA sequestration for its delayed translation (that in some cases takes place several days, or even weeks, after transcription), are viewed as a strategy to regulate the time of synthesis for proteins required in the transcriptionally inert elongating and elongated spermatids. The mRNAs for transition proteins and protamines themselves, although attaining their expression peak in RS, would start to be transcribed as early as in P/D (da Cruz et al., 2016), thus being stored for weeks before translation. Moreover, it is known that their premature translation is related to spermiogenesis arrest and infertility (Lee et al., 1995; Tseden et al., 2007). Furthermore, contrasting the results from proteomic studies with those of transcriptomic studies also supports the widespread translational repression in PS (Gan et al., 2013a).

Meiotic and early post-meiotic cells have developed diverse regulatory mechanisms to achieve these unusually high levels of post-transcriptional regulation (Kleene, 2001, 2013). These mechanisms include binding of repressor proteins (e.g., Idler and Yan, 2012) and sequestration of mRNAs as free ribonucleoprotein particles (Iguchi et al., 2006); manipulation of the length of 3’UTRs (Li et al., 2016); regulation through sncRNAs (Yadav and Kotaja, 2014) and lncRNAs (see below): sequestration of mRNAs in the chromatoid body of post-meiotic cells (Meikar et al., 2011, 2014; de Mateo and Sassone-Corsi, 2014; Lehtiniemi and Kotaja, 2018), and others. Particularly, an RNAseq study of purified mouse male germ cells indicates that coordinated intron retention is a mechanism of male meiotic cells to produce stable, long-lived transcripts that are preserved for days before their timely translation in transcriptionally inert post-meiotic cells. Moreover, intron-retention genes were specifically enriched in functional categories related to the late spermiogenic phase, thus revealing a meiosis-specific mechanism for the uncoupling between transcription and translation (Naro et al., 2017).

Finally, in the recent years an important role for post-transcriptional regulation has been also revealed at the transition from mitotic divisions to meiotic program, both in males and females, which would take place through a complex of MEIOC (Abby et al., 2016; Soh et al., 2017) and the RNA helicase YTHDC2 (Bailey et al., 2017; Hsu et al., 2017; Wojtas et al., 2017; Jain et al., 2018). Diverse approaches including microarrays (Abby et al., 2016) and different RNAseq studies (Bailey et al., 2017; Hsu et al., 2017; Soh et al., 2017; Wojtas et al., 2017; Jain et al., 2018) suggest that this – in turn mediated via a complex between the meiotic initiator MEIOSIN and STRA8 (Ishiguro et al., 2020) – would be accomplished by controlling mRNA stability, either stabilizing meiotic transcripts (Abby et al., 2016), destabilizing transcripts of mitotic cell cycle genes (Bailey et al., 2017; Hsu et al., 2017; Soh et al., 2017; Wojtas et al., 2017), or maybe both (Jain et al., 2018).

## Meiotic Long Non-Coding RNAs (lncRNAs)

An RNAseq study indicated that the testes exhibit substantially higher expression of both genic and intergenic sequences than any other organ, in different mammalian species (Soumillon et al., 2013). Moreover, this widespread testicular transcription is especially prevalent in meiotic spermatocytes and post-meiotic RS, and has been proposed to be a consequence of the extensive chromatin remodeling, which would promote a permissive chromatin state (Soumillon et al., 2013). Remarkably, this higher expression in testis is especially notorious for lncRNAs (Cabili et al., 2011; Derrien et al., 2012; Soumillon et al., 2013; Necsulea et al., 2014; Hong et al., 2018; Darbellay and Necsulea, 2020); therefore, here we will specifically focus on lncRNAs.

By definition, lncRNAs are RNAs longer than 200 nucleotides (as opposed to sncRNAs) that lack protein-coding potential (Kapranov et al., 2007; Atkinson et al., 2012; Rinn and Chang, 2012), despite some of them might actually encode short functional peptides (Li et al., 2017). They are mostly transcribed by RNA polymerase II, in general show lower expression levels than coding genes, tend to be lowly conserved, and exhibit high tissue- and developmental-specific expression patterns (Cabili et al., 2011; Derrien et al., 2012; Ulitsky and Bartel, 2013; Necsulea et al., 2014; Quinn and Chang, 2016). LncRNAs have been implicated in the regulation of diverse biological processes (Ma et al., 2013; Fatica and Bozzoni, 2014; Wu and Du, 2017; Kopp and Mendell, 2018; Barman et al., 2019; Yao et al., 2019), and some of them have been related to the development of different diseases (Wu and Du, 2017; Sanchez Calle et al., 2018; Barman et al., 2019; Tsagakis et al., 2020), including ovarian and testicular pathologies (Lü et al., 2015; Liu et al., 2017; Das et al., 2018; Li et al., 2018; Zhang et al., 2018; Salemi et al., 2019).

Given its high numbers of expressed lncRNAs, the testis is an ideal system for their study. Moreover, it is believed that at least a subset of them may play important regulatory roles in spermatogenesis (e.g., Luk et al., 2014). A few transcriptomic studies have identified and partially characterized lncRNAs to variable extents in isolated spermatogenic cell types through microarrays (Liang et al., 2014) or strand-specific RNAseq (essential for the accurate identification of antisense lncRNAs), in mouse (Soumillon et al., 2013; Lin et al., 2016; Wichman et al., 2017; Chen et al., 2018; Trovero et al., 2020) and human (Rolland et al., 2019). Most of these studies reported the highest lncRNA numbers in meiotic, and mainly in post-meiotic cells (Soumillon et al., 2013; Lin et al., 2016; Chen et al., 2018; Rolland et al., 2019; Trovero et al., 2020). Interestingly, Chen et al. (2018), whose scRNAseq profiling of discrete cell subtypes was the only study to include D and metaphase I, found very high expression levels of lncRNAs in those cells, which allows to suspect that a high number of lncRNAs might be upregulated at those late meiotic stages.

Concerning meiotic lncRNAs, curiously Chalmel et al. (2014) reported that in rat they are longer than those differentially expressed in other stages due to greater exon length, and this was also reported for human (Rolland et al., 2019), although it was not corroborated for mouse (Trovero et al., 2020).

At least some of the meiotic lncRNAs would most probably be involved in the regulation of meiotic processes, despite few examples are available thus far. One such examples is *Mrhl* (*meiotic recombination hot-spot locus*), which is downregulated in spermatogonia upon the activation of the Wnt-signaling pathway (Arun et al., 2012). *Mrhl* inactivation would cause its release from the promoter of *Sox8* (that encodes a developmentally important transcription factor), thus leading to *Sox8* upregulation and the expression of genes required for meiotic commitment (Kataruka et al., 2017). LncRNA *Tesra*, which is transcribed from the *Prss/Tessp* locus mainly (although not exclusively) in PS, has been shown to bind the promoter of *Prss42/Tessp-2* – that encodes an important serine-protease for meiotic progression – and increase its activity (Satoh et al., 2019). *Gm2044*, which is transcriptionally activated by A-MYB (see above), has been shown to regulate the expression of the synaptonemal complex component-coding gene *Sycp1* by acting as a microRNA-sponge in mouse spermatocyte-derived GC-2 cells (Liang et al., 2020). *R53*, a SINE-containing lncRNA, is associated to meiotic metaphase chromatin and apparently would play an indispensable role for spermatogenesis progression, as its knockdown revealed a remarkable reduction of post-meiotic cells and irregular upregulation of several post-meiotic genes (Nakajima et al., 2017). Similarly, a role for meiosis progression has been suggested for *Tsx*, an X-linked lncRNA differentially expressed in PS, as knockout mice show increased PS apoptosis (Anguera et al., 2011). On the other hand, *2193* lncRNA has been identified by means of scRNAseq, and shown to play an important role in porcine oocyte maturation through epigenetic modification (Yang et al., 2020).

Of special interest, Ding et al. (2012, 2019) identified lncRNAs that accumulate at their respective gene *loci* in the fission yeast *Schizosaccharomyces pombe*, and mediate homologous recognition and robust pairing during meiotic prophase. So far, we ignore whether a similar mechanism may be operative in mammalian meiosis. No doubt, the years to come will shade light on the roles of lncRNAs, including their participation in the regulation of the unique meiotic processes.

## Meiotic Sex Chromosome Inactivation (MSCI) Viewed From the Transcriptomic Perspective

Meiotic sex chromosome inactivation is the epigenetic silencing of the sex chromosomes that takes place in male mammals during meiotic prophase I. It is considered to avoid recombination between non-homologous regions of the sex chromosome pair, and is followed by partial transcriptional reactivation in RS (reviewed in Yan and McCarrey, 2009; Checchi and Engebrecht, 2011; Sin and Namekawa, 2013; Lu and Yu, 2015; Turner, 2015; Daish and Grützner, 2019).

**FIGURE 2:**
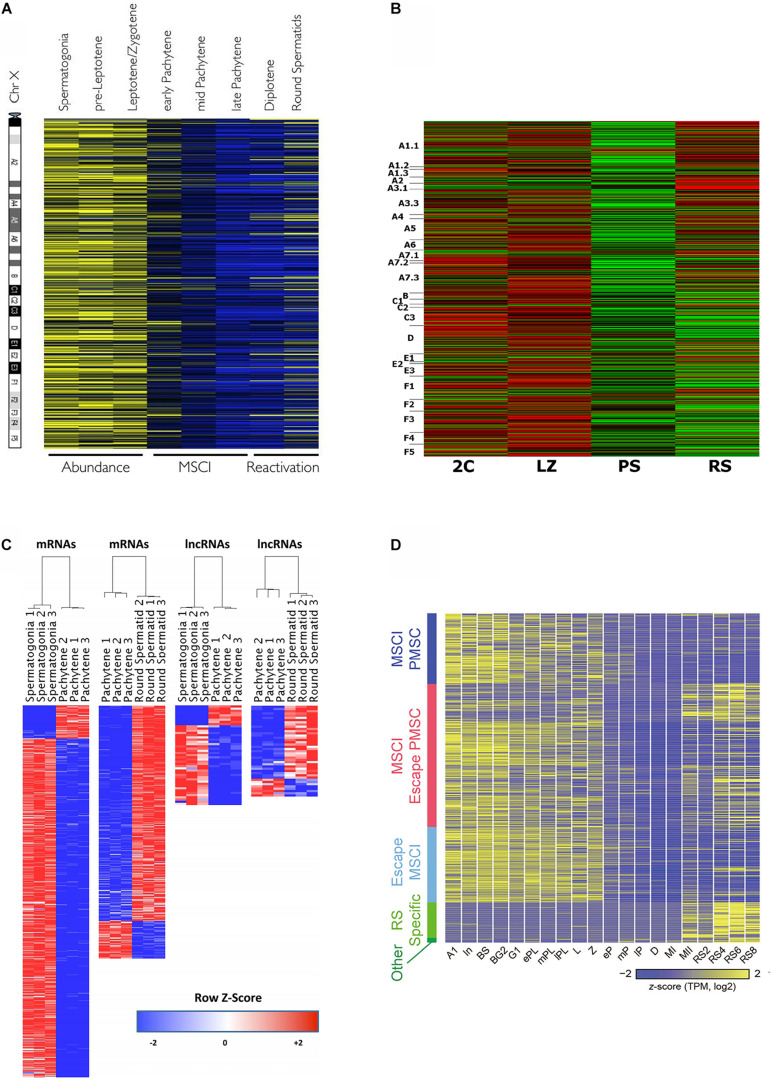
Heat maps from different transcriptomic studies evidencing MSCI in mouse. **(A)** Relative expression levels of 874 X-linked genes, as obtained by microarray analysis, and ordered relative to their chromosome location from centromere to telomere. The different spermatogenic cell populations were purified by FACS. High expression is shown in yellow, and low expression in blue. The figure is reproduced from Fallahi et al. (2010), under the Creative Commons Attribution License. **(B)** Relative expression levels of X-linked protein-coding genes for FACS-sorted testicular cell populations, resulting from RNAseq analysis (cell-type abbreviations are the same as in Figure 1A,c). The genes are ordered according to their position on the chromosome from *p* to *q*. High expression: red; low expression: green. The figure is reproduced from da Cruz et al. (2016), under the Creative Commons Attribution License. **(C)** Differentially expressed X- and Y-linked mRNAs and lncRNAs between spermatogonia vs. PS, and PS vs. RS, on a red-to-blue scale. The cell populations were purified by STA-PUT, and transcriptomic profiles were obtained through RNAseq. Reprinted from Wichman et al. (2017), with permission from Oxford University Press (license number 4933230295359). **(D)** Expression patterns of sex chromosome-linked genes along 20 developmental stages, obtained through scRNAseq of synchronized spermatogenic cells (Concerning meiotic cells, L, leptotene; Z, zygotene; eP, early pachytene; mP, middle pachytene; lP, late pachytene; D, diplotene; MI, metaphase I; MII, metaphase II). The genes are grouped according to: MSCI PMSC (post-meiotic sex chromatin silencing), MSCI/escape PMSC, escape MSCI, RS-specific, and other. Colors from yellow to blue represent high to low relative expression levels. The figure is reproduced from Chen et al. (2018), under the Creative Commons Attribution License.

Massive gene expression analyses allow to visualize MSCI in a very graphical way (Figure 2), and hence most transcriptomic studies along spermatogenesis have evaluated the dynamics of X chromosome inactivation and reactivation (Namekawa et al., 2006; Fallahi et al., 2010; Soumillon et al., 2013; Margolin et al., 2014; Sin et al., 2015; Ball et al., 2016; da Cruz et al., 2016; Wichman et al., 2017; Chen et al., 2018; Green et al., 2018; Lukassen et al., 2018; Ernst et al., 2019; Grive et al., 2019; Jung et al., 2019; Shami et al., 2020), which in turn is useful as a means to confirm the robustness of the employed cell-type classification methods and the reliability of the obtained transcriptomic data.

All the above-cited reports agree that gene expression from the sex chromosomes is massively silenced during the P stage. However, while it is accepted that MSCI in primates is less complete than in mice (de Vries et al., 2012; Shami et al., 2020), controversy exists about the extent of mouse MSCI. A number of transcriptomic studies have reported that a few X-linked mRNAs with high expression levels during late meiotic prophase I, would escape MSCI (Soumillon et al., 2013; da Cruz et al., 2016; Wichman et al., 2017; Chen et al., 2018). Contradicting these findings, a couple of recent scRNAseq analyses did not detect MSCI escapees during P/D in mouse (Jung et al., 2019; Shami et al., 2020). On the other hand, it is fairly accepted that a subset of microRNAs escape MSCI (Song et al., 2009; Sosa et al., 2015), albeit this has been controverted as well (Royo et al., 2015). Concerning lncRNAs, although no enough information is available yet, it has been suggested that X- and Y-linked lncRNAs would be mostly subject to MSCI, with a few of them escaping inactivation (Wichman et al., 2017).

In relation to MSCI and the partial post-meiotic reactivation (e.g., Namekawa et al., 2006; Mueller et al., 2008), an interesting finding is the establishment of active epigenetic marks on enhancers and promoters of silent sex chromosomes during meiosis, which would act as epigenetic memory, poising genes for their subsequent activation in RS (Sin et al., 2012; Adams et al., 2018). This would probably be in agreement with a previous observation that specific regions of the sex chromosomes during P retain active chromatin marks (Khalil and Driscoll, 2007). On the other hand, Ernst et al. (2019) reported that in PS, the promoters of spermatid-specific genes on the X chromosome carry high levels of the repressive chromatin mark H3K9me3, which would decrease in RS (observing at least for an analyzed example, a bivalent chromatin state), and suggested that these high H3K9me3 levels could be necessary to prevent premature transcription of X-linked spermatid-specific genes.

In addition, it has been reported that in spite of transcriptional silencing, MSCI would be accompanied by the massive *de novo* formation of accessible chromatin in the sex chromosomes in PS (Maezawa et al., 2018). In fact, genome-wide chromatin studies (Hi-C, ChIP-seq), some of them in combination with RNAseq, reveal a distinct higher-order chromatin structure in the sex chromosomes during MSCI (Alavattam et al., 2019; Patel et al., 2019; Vara et al., 2019; Wang et al., 2019).

## Conclusion

Genome-wide characterization of the gene expression programs underlying the unique events that take place along meiotic prophase, and how they are regulated, is essential for the comprehensive understanding of meiosis groundwork. Deepening the knowledge of this extraordinary and highly complex process is indispensable for the development of therapeutic approaches, as the alteration of meiotic events is at the base of numerous pathologies including a high number of idiopathic infertility cases. Methodological advances to allow the analyses of specific cell types among the complex testicular tissue, together with modern omics techniques, provide a broad picture, while starting to disclose a detailed molecular landscape of the different stages of spermatogenesis in mouse, and also in human. These studies, carried out through different approaches and platforms, have often reached some confluent results, and revealed that meiotic spermatocytes, as well as post-meiotic spermatids, have highly complex transcriptomes. Here, we have focused on some of these results, and particularly concerning meiotic prophase gene expression. In synthesis, we highlight that despite early meiotic prophase cells have lower overall expression levels and a less complex transcriptome than other spermatogenic cell types, they differentially express a set of genes related to male meiosis, and in a few cases even to post-meiosis. We point out that transcriptomic analyses allow to appreciate the real magnitude of post-transcriptional regulation and translational delay along the process, showing their massiveness, as well as the switch in gene expression programs during meiotic prophase (particularly the P stage); in turn, this would be accompanied by extensive and sophisticated regulatory mechanisms to guarantee the perfect execution timing of the spermatogenic programs, which is essential for the production of healthy sperm. Transcriptomic studies have also provided an easy way to visualize massive meiotic-specific processes such as MSCI, and compare its extension between different species. Besides, transcriptomic studies have revealed the existence of a higher number of lncRNAs in spermatogenic cells than in any other analyzed cell type or tissue. Important regulatory roles are starting to be revealed for some of these lncRNAs in relation to meiosis, although, no doubt, we have only just begun to see the tip of the iceberg.

## Author Contributions

AG wrote most of the manuscript. RR-C and RB participated in the design, conception and writing, corrected the manuscript, and approved it for publication. All authors contributed to the article and approved the submitted version.

## Conflict of Interest

The authors declare that the research was conducted in the absence of any commercial or financial relationships that could be construed as a potential conflict of interest.
